# Community-acquired MRSA and pig-farming

**DOI:** 10.1186/1476-0711-5-26

**Published:** 2006-11-10

**Authors:** Xander W Huijsdens, Beatrix J van Dijke, Emile Spalburg, Marga G van Santen-Verheuvel, Max EOC Heck, Gerlinde N Pluister, Andreas Voss, Wim JB Wannet, Albert J de Neeling

**Affiliations:** 1 National Institute for Public Health and the Environment (RIVM), Diagnostic Laboratory for Infectious Diseases and Perinatal Screening, P.O. Box 1, 3720 BA, Bilthoven, The Netherlands; 2 St. Jansgasthuis, Department of Medical Microbiology, P.O. Box 29, 6000 AA, Weert, The Netherlands; 3 Radboud University Medical Centre, Department of Medical Microbiology, P.O. Box 9101, 6500 HB, Nijmegen, The Netherlands; 4 Canisius-Wilhelmina Hospital, Department of Medical Microbiology, P.O. Box 9015, 6500 GS, Nijmegen, The Netherlands

## Abstract

**Background:**

Sporadic cases of CA-MRSA in persons without risk-factors for MRSA carriage are increasing.

**Case presentation:**

We report a MRSA cluster among family members of a pig-farmer, his co-workers and his pigs. Initially a young mother was seen with mastitis due to MRSA. Six months later her baby daughter was admitted to the hospital with pneumococcal otitis. After staying five days in hospital, the baby was found to be MRSA positive. At that point it was decided to look for a possible source, such as other family members and house-hold animals, including pigs on the farm, since those were reported as a possible source of MRSA earlier.

Swabs were taken from the throat and nares of family members and co-workers. A veterinarian obtained swabs from the nares, throat and perineum of 10 pigs. Swabs were cultured following a national protocol to detect MRSA that included the use of an enrichment broth. Animal and human strains were characterized by PFGE, *spa*-typing, MLST analysis, SSC*mec*, AGR typing, and the detection for PVL, LukM, and TSST toxin genes.

Three family members, three co-workers, and 8 of the 10 pigs were MRSA positive. With the exception of the initial case (the mother) all persons were solely colonized, with no signs of clinical infections.

After digestion with *Sma*I, none of the strains showed any bands using PFGE. All isolates belonged to *spa *type t108 and ST398.

**Conclusion:**

1. This report clearly shows clonal spread and transmission between humans and pigs in the Netherlands. 2. MLST sequence type 398 might be of international importance as pig-MRSA, since this type was shown earlier to be present in epidemiologically unrelated French pigs and pig-farmers. 3. Research is needed to evaluate whether this is a local problem or a new source of MRSA, that puts the until now successful Search and Destroy policy of the Netherlands at risk.

## Background

*Staphylococcus aureus *is a major pathogen causing both nosocomial and community-acquired infections. MRSA strains have emerged worldwide and became resistant to a variety of antibiotics. The prevalence of MRSA varies widely between countries, from less than 1% in the Netherlands to more than 30% in several other European countries [1]. Bacterial strain typing is an important tool to investigate MRSA outbreaks, to evaluate the transmission of MRSA strains, and to study evolution. PFGE with *Sma*I is considered to be the gold standard for molecular typing of MRSA [2]. When no *Sma*I digestion occurred, MRSA strains were classified as non-typeable by PFGE. Recently, Voss and colleagues described a possible link between non-typeable MRSA and pig farming [3]. French farmers were shown to be colonized by a small number of *S. aureus *strains which exhibited MLST sequence types (ST) 9, 398, and 433. These STs were found in isolates from pig farmers as well as from swine but were not present in non-farmers suggesting a high rate of *S. aureus *strain exchange between pig farmers and pigs had occurred [4]. MRSA of animal origin may be genetically related to MRSA recovered from humans [5]. MRSA in companion animals have also been described as source for infection for animals and humans [6,7].

The aim of this study was to find the source of MRSA in a family of a pig-farmer that had no known risk-factors for MRSA carriership, but were found to be permanent carriers of PFGE non-typeable MRSA.

## The case

In October 2004 a young mother with mastitis suffering from high fevers (> 39°C), general malaise, and pleural effusions, was admitted to our hospital. Cultures taken at her GP's office unexpectedly revealed MRSA. The patient recovered quickly after treatment with teicoplanin. When repeated attempts to eradicate her MRSA carriership failed, her family was screened for MRSA. The father and the baby daughter were found to be MRSA positive. Six month later, the baby girl was admitted with an acute pneumococcal infection. Due to the history of MRSA the baby was isolated and screened on admission. While initial screening cultures were negative, follow-up cultures during antibiotic treatment revealed MRSA. At this point all family members were re-screened and the parents were found to still carry MRSA. The source of MRSA remained unclear. As animals have been described as a source of MRSA and the father was a pig-farmer, we decided to screen his pigs. Furthermore, three co-workers on the farm were screened.

The farm consisted of 8000 pigs located in 4 different holdings. We randomly picked 10 pigs from the holding closest to the living quarters of the family. A veterinarian took cultures from the anterior nares, throat and perineum of the animals. All cultures were processed in the laboratory according to a national guideline for the detection of MRSA in human samples. Swabs were put into an enrichment broth that was incubated for 24 hours at 37°C and subcultured on blood agar. The cefoxitin disc method was used to screen for methicillin-resistance in colonies suspected to be *S. aureus*.

Identification of MRSA was confirmed by a multiplex PCR in which a *S. aureus *specific DNA fragment [8] and the *mecA *gene for methicillin resistance [9] is amplified. Oxacillin susceptibility was tested by E-test (AB Biodisk) on Mueller-Hinton agar (BBL) containing 2% NaCl with 24 h incubation at 35°C and results were interpreted according to the criteria of the Clinical and Laboratory Standards Institute [10]). In bovine mastitis, the leukocidin LukM is considered to be a virulence factor [11]. Since the mother of the pig-farming family suffered from mastitis, all non-typeable MRSA isolates were tested for the presence of the *LukM *gene [12]. The presence of the *tst *gene, encoding for the toxic shock syndrome toxin (TSST), was also investigated [13]. This gene was found significantly more often in mastitis-associated *S. aureus *strains [14]. All PFGE non-typeable MRSA strains were characterized by staphylococcal protein A (*spa*) gene typing [15], multi-locus sequence typing (MLST) [16], staphylococcal chromosome cassette (SCC) *mec *typing [17], accessory gene regulator (AGR) typing [18], and the detection of the Panton-Valentine leukocidin (PVL) genes[19]. PVL is a virulence factor thought to be associated with community-acquired MRSA [19].

Using different typing methods all (animal as well as human) PFGE non-typeable MRSA isolates were shown to be genetically identical. They were characterized by *spa *type t108, ST398, SCC*mec *type V, AGR type 1, and negative for the PVL, LukM and TSST toxins.

## Discussion

The MLST results are in concordance with a study reported by Armand-Lefevre and colleagues, who compared *S. aureus *isolates from healthy pig farmers, human controls, and pigs [4]. They recovered methicillin-susceptible *S. aureus *exhibiting ST9, 398, and 433 from pig farmers and swine; only one ST 398 isolate of a pig farmer was methicillin resistant. ST398 was first recognised by our group, and reported to the international MLST database in 2004. At that time no correlation between *S. aureus *with ST398 and pig farming had been reported. In Hong Kong, two ST398 strains were described to have been isolated from patients with bacteremia [20]. No relation with pig farming was reported. Typing results of the French ST398 strains (4 pig-related MSSA and 1 pig-related MRSA isolate) revealed the same typing result as the Dutch ST398 strains. At our lab the French strains were PFGE non-typeable, *spa *type t034 and t1250, and were PVL negative. Spa type t108, t034, and t1250 are related to each other, indicating to have a common ancestor.

Voss and colleagues reported for the first time the isolation of PFGE non-typeable MRSA strains from pig care-takers [3]. The strains were closely related to each other as shown by *spa *typing. They screened a total of 26 farmers of whom 6 were colonized with MRSA. The authors identified three different MRSA strains by *spa *typing, type t108, t567, and t943. Spa type t108 was also found in the present study, indicating the relatedness of this *spa *type with pig-farming. Only one pig was found to be MRSA positive, carrying the same strain type as the farmer. In contrast, we found MRSA in 8 out of 10 randomly chosen pigs. The difference in prevalence could perhaps be explained by sampling differences, MRSA transmission among pigs or to differences in risk factors between the farms.

All pig MRSA isolates were PFGE non-typeable by PFGE and had the same typing characteristics as the human MRSA isolates. Furthermore, the pig-related MRSA isolates were related to PFGE non-typeable MRSA strains from the national MRSA database. It seems that the PFGE non-typeable MRSA strains are not only transmitted between human and pigs but also between humans. The human to human transmission was elucidated by the fact that among the PFGE non-typeable MRSA isolates from the national institute of public health (RIVM) MRSA database in at least 3 cases a family member was colonized with an MRSA strain with identical typing characteristics. Furthermore, the child of the pig farmer's family had no contact with pigs and was colonized with the same strain as the parents.

An earlier report of a significant association between pig farming and resistant commensal bacteria was published by Aubry-Damon et al. [21]. The authors showed that levels of commensal bacteria with antimicrobial resistance were higher among pig farmers than among controls, including a higher isolation rate of *S. aureus *in pig farmers. The cause of the higher *S. aureus *isolation rate in pig farmers remained unclear.

More research on a larger scale is necessary to further address the prevalence of MRSA among pigs, pig farmers and their contacts. Furthermore, it would be interesting to what extent the PFGE non-typeable MRSA isolates were associated with pig farming, which may elucidate the importance of the clonal cluster.

## Conclusion

This report clearly shows the clonal spread and transmission between man and pigs in the Netherlands. MRSA isolates characterized by *spa *type t108 (or related *spa *types) and MLST ST 398 might be of international importance as pig-MRSA, since this type was shown earlier to be present among epidemiological unrelated MRSA isolates from French pigs and pig-farmers. Further research has to evaluate whether pigs are a new source of MRSA, that warrants a change in the Search & Destroy strategy, namely by adding pig-farmers pigs to the group of possible MRSA carriers.

The prevalence of MRSA in farming animals, as well in the humans working with them, (e.g. farmers, veterinarians) needs to be established.

## Competing interests

The author(s) declare that they have no competing interests.

## Authors' contributions

XWH designed the study, collected and analyzed the data and drafted the manuscript. ES, MGS, MEOCH, GNP performed experimental work. BJD and AV were involved in the pig-MRSA related case. WJB and AJN participated in the design of the study and drafting of the manuscript. All authors read and approved the final manuscript.

## References

[B1] Tiemersma EW, Bronzwaer SL, Lyytikainen O, Degener JE, Schrijnemakers P, Bruinsma N, Monen J, Witte W, Grundman H (2004). Methicillin-resistant *Staphylococcus aureus* in Europe, 1999-2002. Emerg Infect Dis.

[B2] Murchan S, Kaufmann ME, Deplano A, de Ryck R, Struelens M, Zinn CE, Fussing V, Salmenlinna S, Vuopio-Varkila J, El Solh N, Cuny C, Witte W, Tassios PT, Legakis N, van Leeuwen W, van Belkum A, Vindel A, Laconcha I, Garaizar J, Haeggman S, Olsson-Liljequist B, Ransjo U, Coombes G, Cookson B (2003). Harmonization of pulsed-field gel electrophoresis protocols for epidemiological typing of strains of methicillin-resistant *Staphylococcus aureus*: a single approach developed by consensus in 10 European laboratories and its application for tracing the spread of related strains. J Clin Microbiol.

[B3] Voss A, Loeffen F, Bakker J, Klaassen C, Wulf M (2005). Methicillin-resistant *Staphylococcus aureus* in pig farming. Emerg Infect Dis.

[B4] Armand-Lefevre L, Ruimy R, Andremont A (2005). Clonal comparison of *Staphylococcus aureus* isolates from healthy pig farmers, human controls, and pigs. Emerg Infect Dis.

[B5] Lee JH (2003). Methicillin (Oxacillin)-resistant *Staphylococcus aureus* strains isolated from major food animals and their potential transmission to humans. Appl Environ Microbiol.

[B6] van Duijkeren E, Wolfhagen MJ, Heck ME, Wannet WJ (2005). Transmission of a Panton-Valentine leucocidin-positive, methicillin-resistant *Staphylococcus aureus* strain between humans and a dog. J Clin Microbiol.

[B7] Baptiste KE, Williams K, Willams NJ, Wattret A, Clegg PD, Dawson S, Corkill JE, O'Neill T, Hart CA (2005). Methicillin-resistant staphylococci in companion animals. Emerg Infect Dis.

[B8] Martineau F, Picard FJ, Roy PH, Ouellette M, Bergeron MG (1998). Species-specific and ubiquitous-DNA-based assays for rapid identification of *Staphylococcus aureus*. J Clin Microbiol.

[B9] De Neeling AJ, van Leeuwen WJ, Schouls LM, Schot CS, Veen-Rutgers A, Beunders AJ, Buiting AG, Hol C, Ligtvoet EE, Petit PL, Sabbe LJ, van Griethuysen AJ, van Embden JD (1998). Resistance of staphylococci in The Netherlands: surveillance by an electronic network during 1989-1995. J Antimicrob Chemother.

[B10] CLSI (2005). Performance standards for antimicrobial susceptibility testing; 15th informational supplement. CLSI document M100-S15, Wayne, Pennsylvania, USA.

[B11] Younis A, Krifucks O, Fleminger G, Heller ED, Gollop N, Saran A, Leitner G (2005). *Staphylococcus aureus* leucocidin, a virulence factor in bovine mastitis. J Dairy Res.

[B12] Jarraud S, Mougel C, Thioulouse J, Lina G, Meugnier H, Forey F, Nesme X, Etienne J, Vandenesch F (2002). Relationships between *Staphylococcus aureus* genetic background, virulence factors, agr groups (alleles), and human disease. Infect Immun.

[B13] Mehrotra M, Wang G, Johnson WM (2000). Multiplex PCR for detection of genes for *Staphylococcus aureus* enterotoxins, exfoliative toxins, toxic shock syndrome toxin 1, and methicillin resistance. J Clin Microbiol.

[B14] van Leeuwen WB, Melles DC, Alaidan A, Al Ahdal M, Boelens HA, Snijders SV, Wertheim H, van Duijkeren E, Peeters JK, van der Spek PJ, Gorkink R, Simons G, Verbrugh HA, van Belkum A (2005). Host- and tissue-specific pathogenic traits of *Staphylococcus aureus*. J Bacteriol.

[B15] Harmsen D, Claus H, Witte W, Rothganger J, Claus H, Turnwald D, Vogel U (2003). Typing of methicillin-resistant *Staphylococcus aureus* in a university hospital setting by using novel software for spa repeat determination and database management. J Clin Microbiol.

[B16] Enright MC, Day NP, Davies CE, Peacock SJ, Spratt BG (2000). Multilocus sequence typing for characterization of methicillin-resistant and methicillin-susceptible clones of *Staphylococcus aureus*. J Clin Microbiol.

[B17] Zhang K, McClure JA, Elsayed S, Louie T, Conly JM (2005). Novel multiplex PCR assay for characterization and concomitant subtyping of staphylococcal cassette chromosome mec types I to V in methicillin-resistant *Staphylococcus aureus*. J Clin Microbiol.

[B18] Lina G, Boutite F, Tristan A, Bes M, Etienne J, Vandenesch F (2003). Bacterial competition for human nasal cavity colonization: role of Staphylococcal agr alleles. Appl Environ Microbiol.

[B19] Lina G, Piemont Y, Godail-Gamot F, Bes M, Peter MO, Gauduchon V, Vandenesch F, Etienne J (1999). Involvement of Panton-Valentine leukocidin-producing *Staphylococcus aureus* in primary skin infections and pneumonia. Clin Infect Dis.

[B20] Ip M, Yung RW, Ng TK, Luk WK, Tse C, Hung P, Enright M, Lyon DJ (2005). Contemporary methicillin-resistant *Staphylococcus aureus* clones in Hong Kong. J Clin Microbiol.

[B21] Aubry-Damon H, Grenet K, Sall-Ndiaye P, Che D, Cordeiro E, Bougnoux ME, Rigaud E, Le Strat Y, Lemanissier V, Armand-Lefevre L, Delzescaux D, Desenclos JC, Lienard M, Andremont A (2004). Antimicrobial resistance in commensal flora of pig farmers. Emerg Infect Dis.

